# Quality of sleep among trainee doctors at the Charles Nicolle Hospital during the 2nd wave of COVID19

**DOI:** 10.1192/j.eurpsy.2023.2350

**Published:** 2023-07-19

**Authors:** Z. Athimni, I. Youssef, G. Bahri, M. Mersni, D. Brahim, N. Mechergui, N. Ladhari

**Affiliations:** 1 MEDECINE DU TRAVAIL, CENTRE HOSPITALO-UNIVERSITAIRE CHARLES NICOLLE, UNIVERSITE DE TUNIS, TUNIS, Tunisia

## Abstract

**Introduction:**

Sleep quality depends on several factors such as smoking, physical activity, diet, and certain pathologies such as obstructive sleep apnoea syndrome. Indeed, following a COVID-19 infection, several trainee doctors complained about a deterioration of their sleep quality.

**Objectives:**

To evaluate the quality of sleep of medical trainees working at Charles Nicolle Hospital who were infected by SARS-COV2.

**Methods:**

We conducted a descriptive cross-sectional study, among medical trainees at Charles Nicolle Hospital, infected by COVID-19 during the period from July 2020 to November 2020. Sleep quality was evaluated by the Pittsburgh Sleep Quality Index (PSQI) questionnaire. Trainees were contacted during the period August 2022 to September 2022.

**Results:**

Fifty-three trainee doctors have joined our study. Forty-five of them had a significant sleep disturbance with a Pittsburgh Sleep Quality Index (PSQI) greater than five. The average age was 29.4±3.07 years old with a female majority (75%). No psychiatric history was found. The most affected category of trainees were residents (74%), particularly those working in the general surgery department (18%) and the anaesthesia and intensive care department (9%). Among those trainees, 80% had night shifts with an average of six shifts per month. Sleep latency was high in 20% of cases. A sleep duration of less than five hours per night was found in 18% of the cases. Six participants reported using sleeping pill three to four times a week.

**Conclusions:**

Our study revealed a significant sleep disturbance among trainee doctors. This could be due to the SARS-COV2 infection but can also be explained by the night shifts burden and the great mental load at work during this pandemic period.

**Disclosure of Interest:**

None Declared

